# Computational and spectroscopic ‍characterization of thianthrene

**DOI:** 10.1098/rsos.231846

**Published:** 2024-05-01

**Authors:** Rachel H. Rushworth, Matei Pascariu, Mona Sarter, Stewart F. Parker

**Affiliations:** ^1^ ISIS Facility, STFC Rutherford Appleton Laboratory, Chilton, Didcot, OX11 0QX, UK

**Keywords:** thianthrene, inelastic neutron scattering spectroscopy, Raman spectroscopy, infrared spectroscopy

## Abstract

In this work, we have carried out a comprehensive characterization of the vibrational spectroscopy of the non-planar molecule thianthrene. The combination of infrared, Raman and inelastic neutron scattering spectroscopies is highly complementary and allows all of the modes to be observed. Periodic density-functional theory calculations have provided unambiguous assignments of the spectra. The literature states that C–S stretch modes occur in the 600–800 cm^−1^ range. We find that while there are modes that involve sulfur motion in this region, this is a consequence of motion in the *ortho*-phenylene rings. The modes that are driven by the C–S stretches are found in the ~400–500 cm^−1^ range. The C–S–C bending modes occur in the 200–300 cm^−1^ range; these have not been previously characterized.

## 1. Introduction

**Figure 1.**
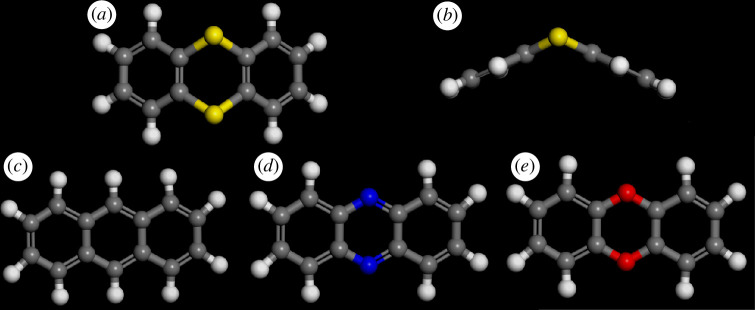
Structure of thianthrene (*a*) plan view, (*b*) side view, (*c*) anthracene, (*d*) phenazine and (*e*) dibenzo-1,4-dioxin.

Thianthrene (9,10-dithiaanthracene; di-*o*-phenylene disulfide (C_6_H_4_S)_2_, see figure 1*a,b* for the structure) was first synthesized in 1869 by Stenhouse who was investigating the dry distillation of sodium benzenesulfonate [1]. Other routes to it include the pyrolysis of diphenyl disulfide [2], the direct reaction of benzene with S_2_Cl_2_ in the presence of AlCl_3_ [3] and the reaction between *o*-dichlorobenzene and hydrogen sulfide at 550°C [4].

In its neutral state, thianthrene is a butterfly-shaped molecule in the gas, solution and solid states, with a dihedral angle of 131° [5], 142° [6] and 128° [7], respectively, between the planes of the two benzene rings (figure 1*b*). This is in marked distinction to its counterparts with C, N, O as bridging atoms (anthracene, phenazine and dibenzo-1,4-dioxin; figure 1*c*–*e*), all of which are planar. However, the selenium derivative, selenanthrene, is also bent in the solid state with a dihedral angle of 123° [8], similar to that of thianthrene. A computational study [9] using natural bond analysis suggested that the planar conformation of dibenzo-1,4-dioxin was caused by the more effective overlap of the oxygen 2*p*_z_ orbital with the π*CC orbitals of the phenylene rings, as compared to the overlap of the 3p_z_–π*CC orbitals in thianthrene and selenanthrene. Thianthrene undergoes facile inversion with an energy barrier of 24–30 kJ mol^−1^ [10].

Thianthrene derivatives have several uses [11,12]. 2,7-Dimethylthianthrene is a key component of shampoos and soaps used to control seborrhoea, pediculosis, scabies and pruritus. It is also used as a cosmetic for the removal of skin freckles. 2,3,7,8-Tetrachlorothianthrene is useful as a co-catalyst with AlCl_3_ or SbCl_5_ to encourage *p*-chlorination of toluene. Thianthrene’s redox properties have been extensively studied. On oxidation, both the radical cation [13] and the dication [14] become almost planar with a dihedral angle of ~180°. The readily reversible oxidation and reduction of thianthrene and its ions have led to potential applications of derivatives in organic redox flow batteries [15].

The solid-state photophysical properties of thianthrene are of interest as a result of the high triplet formation yield and strong phosphorescent properties [16–18]. However, the spectroscopic properties have not been studied; in particular, the modes associated with the sulfur atoms and the butterfly motion are of interest for energy dispersal. In the present work, we provide a comprehensive assignment of the solid-state vibrational spectra of thianthrene from a combination of infrared, Raman and inelastic neutron scattering (INS) spectroscopies. The assignments are supported by density-functional theory (DFT) calculations of the complete unit cell.

## 2. Experimental section

### 2.1. Material and methods

Thianthrene (97%) was purchased from Aldrich and used as received. The transition temperatures of thianthrene (~5.7 mg) were studied over a temperature range of 123–648 K at rates of 2, 5 and 10 K min^−1^ using a Mettler Toledo DSC1 differential scanning calorimeter (DSC). UV–vis spectra were recorded using a Shimadzu UV-2600i spectrometer in the range 800–200 nm. Infrared spectra were measured at 290 K using a Bruker^®^ VERTEX 70 version Fourier transform (FT) infrared spectrometer and a Bruker Diamond ATR accessory. Spectra at 200 K were measured using a SpecAc low-temperature Golden Gate accessory. Spectra were recorded at 4 cm^−1^ resolution with either 64 (290 K) or 256 (200 K) scans and 8 × zero filling to improve the peak shape. The spectra were corrected for the wavelength-dependent path length of ATR using the Bruker software. FT-Raman spectra were recorded at room temperature using a Bruker MultiRam FT-Raman spectrometer with 500 mW laser power at 1064 nm. Resolutions of 4 cm^−1^ (16 scans) and 1 cm^−1^ (64 scans) were used with 8 × zero filling. Variable temperature (300 K – 6 K) dispersive Raman spectra were recorded using 785 nm excitation with a modified Renishaw inVia spectrometer, which has been previously described [19]. The INS spectrum was measured at ~10 K using TOSCA [20,21] at ISIS [22].

### 2.2. Computational studies

Dispersion-corrected (DFT-D) periodic calculations were carried out using the most recent determination [7] as the initial structure with the plane wave pseudo-potential method as employed in the CASTEP code (version 22.11) [23]. Exchange and correlation were approximated using the Perdew-Burke-Ernzerhof (PBE) functional with the Tkatchenko–Scheffler dispersion correction scheme within the generalized gradient approximation, on-the-fly-generated norm-conserving pseudo-potentials were used. The plane wave cut-off energy was 1020 eV and the Brillouin zone sampling of electronic states used a 4 × 6 × 4 Monkhorst–Pack grid (24 k points). The equilibrium structure was converged to |0.0038| eV Å^−1^. Brillouin zone Γ-point phonon transition energies were obtained by diagonalization of dynamical matrices computed using density-functional perturbation theory [24]. These were also used to compute the dielectric response and the Born effective charges, from which the mode oscillator strength tensor and infrared absorptivity were calculated. For the isolated molecule calculations, one molecule of thianthrene was placed at the centre of a 20 × 20 × 12 Å cell in space group *Pmm*2. This ensures that *C*_2v_ symmetry is imposed. The same parameters were used for the geometry optimization as for the experimental cell, except that electronic sampling was only done at the Γ-point in the Brillouin zone. In both systems, the atomic displacements in each mode that are part of the CASTEP output enable mode assignment by visualization of the modes in Materials Studio [25] and are also all that is required to generate the INS spectrum using AbINS [26]. It is emphasized that for all the calculated spectra shown, the transition energies have *not* been scaled.

## 3. Results and discussion

From an initial DSC scan from 123 to 648 K at 10 K min^−1^, no transitions were seen before the melting point around 429 K (see electronic supplementary material, figure S1, and a more detailed discussion). This is in agreement with a heat capacity study that found no transitions over the range 5–550 K [27] and a single crystal X-ray diffraction (XRD) study that found the structures at 163 and 295 K to be the same, apart from the expected lattice expansion with increasing temperature [7].

In the solid state, thianthrene crystallizes in the monoclinic space group *P*2_1_/*c* (no. 14) with four molecules in the primitive cell [7]. Each molecule is on a *C*_1_ site, although the molecular symmetry is approximately *C*_2v_. The presence of four molecules means that each mode of the isolated molecule has four components in the crystal; the crystallographic centre of symmetry results in two being allowed in the infrared and two in the Raman spectra. Note that *all* modes are allowed in the INS spectrum.

**Figure 2.**
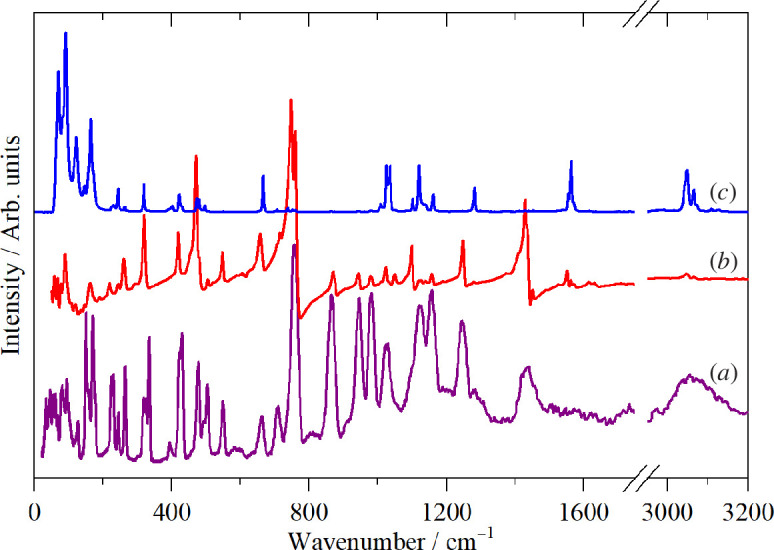
Vibrational spectra of thianthrene (*a*) INS at 10 K, (*b*) FT-Raman at room temperature (1 cm^−1^ resolution) and (*c*) infrared at room temperature.

Figure 2 shows the room temperature infrared and Raman spectra and the 10 K INS spectrum. The room temperature data are preferred because they have the widest range (infrared, 50–4000 cm^−1^) or highest resolution (FT-Raman, 1 cm^−1^) available to us. The complementarity of the three techniques is evident: modes that are weak or absent in the infrared and Raman spectra often appear with good intensity in the INS. In contrast, the C–H stretch region of the INS spectrum (for reasons explained elsewhere [28]) shows no useful information, whereas the infrared and Raman spectra present well-resolved bands.

**Figure 3.**
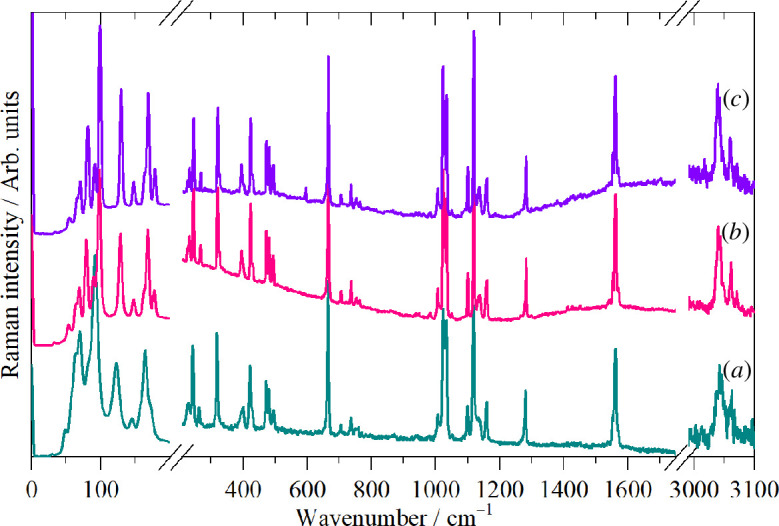
Variable temperature dispersive Raman spectra (785 nm excitation) of thianthrene at (*a*) room temperature, (*b*) 90 K and (*c*) 6 K. The region 200–1750 cm^−1^ is ordinate expanded ×4 and the 2950–3200 cm^−1^ region is ordinate expanded ×40 with respect to the 0–200 cm^−1^ region.

Variable temperature infrared (electronic supplementary material, figure S2) and dispersive Raman (figure 3) spectra show no evidence of any phase changes, in agreement with the heat capacity [27] and XRD [7] studies. While the 6 K Raman spectrum is better resolved, especially in the lattice mode region below 200 cm^−1^, crucially, there are no additional modes present, showing that the room temperature structure is maintained down to at least 6 K.

**Figure 4.**
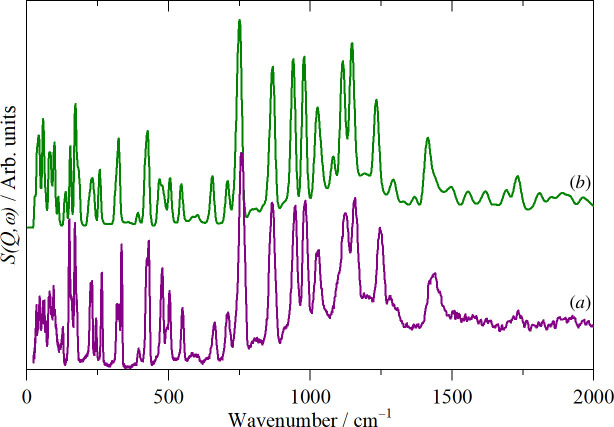
Comparison of (*a*) observed and (*b*) calculated INS spectra of thianthrene.

**Figure 5.**
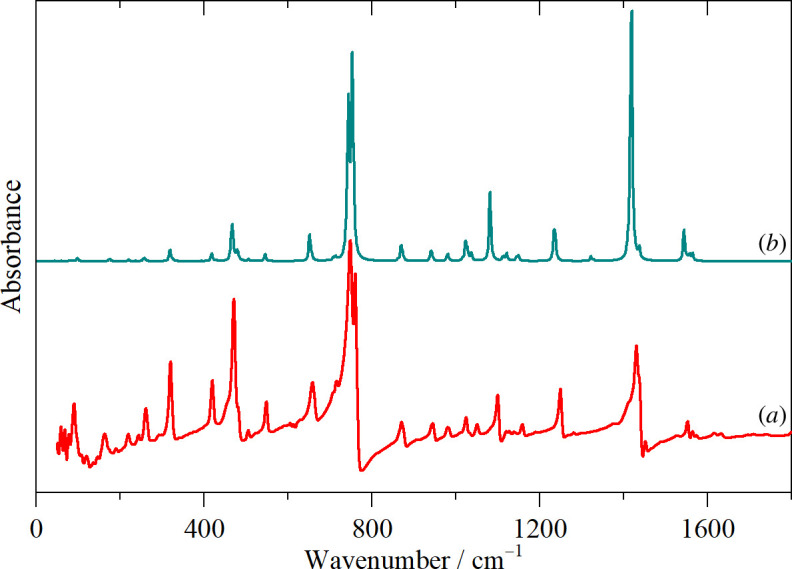
Comparison of (*a*) observed and (*b*) calculated infrared spectra of thianthrene.

It is apparent that the spectra conform to the usual expectations [29]: aromatic C–H stretch >3000 cm^−1^, aromatic C–C stretch 1500–1600 cm^−1^, in-plane C–H bend 1000–1200 cm^−1^, out-of-plane C–H bend 600–800 cm^−1^, ring deformations 300–500 cm^−1^. However, to go beyond these simplistic group frequency assignments, we have carried out DFT calculations of the complete unit cell. A comparison of the observed and calculated INS spectra is shown in figure 4 and the infrared spectra in figure 5. In both cases, it can be seen that the agreement is essentially quantitative.

The combination of infrared, Raman and INS spectra, together with the *ab initio* calculations, enable a complete assignment of the spectra to be made. Thianthrene can be described as two *ortho*-disubstituted phenylene groups linked by sulfur atoms. A consistent set of assignments of *ortho*-disubstituted phenylene groups has been developed by Wright and co-workers [30] and we have adopted their assignments and notation. The form of the modes is reproduced from Tuttle *et al*. [30] in electronic supplementary material, figure S3. Table 1 lists the observed modes and their descriptions, a complete assignment is given in electronic supplementary material, table S1.

**Table 1.** Observed modes of thianthrene, their descriptions and mode assignments.

experimental			assignment^a^
INS^b^ (cm^−1^)	infrared^c^ (cm^−1^)	Raman^d^ (cm^−1^)	
34 w		30 vw (34 vw)	libration
45 w			libration
57 w	59 w	49 w (55 w)	libration
62 w		58 sh (66 w)	libration
69 w	68 w	64 sh (71 s)	translation
78 w	78 w		translation
81 w		70 s (82 s)	libration
89 w			libration
95 w	90 m	82 sh (92 w)	D21(1)
100vw		92 vs (100vs)	libration
128 w	119 vw	123 vs (130vs)	libration
151 s	146 vw	146 w (148 w)	D30
158 m		157 sh (164 w)	D30
171 s	164 m	165 vs (169vs)	D30
178 sh		173 sh (178 m)	D30
226 m/231 m	220 w	226 sh/230 w	D29
245 m	244 w	244 m	in-phase C–S–C bend (butterfly)
264 m	261 m	264 w	D20
318 m/326 m	320 s	319 m	D29
335 s			D20
395 w		391 sh/402 w	D19
425 m/431 s	419 m	423 m/431 w	D28
	471 s	474 m	D19
479 s	480 w	482 m	D18
495 w/504 m	505vw	498 w	D27
550 m	549 m	552 vw	D27
663 m	658 m	666 m	D17
712 m	707 vw/715 vw	707 w	D26
759 vs	748 vs, 760 vs	752 w, 761 w	D25
867 vs, br	870 w, 876 sh	863 vw/866 vw	D24
		874 vw	D24
946 s	945 w	940 vw, 947vw	D23
983 s	982 w, br	983 w	D22
1028 m, br	1024 w	1026 m	D15
		1032 m, 1037 m	D15
	1050 w	1055vw	D12
1090 sh	1100 m	1103 w	D10
1124 s	1120 w, 1127 w	1122 m	D10
	1140 w	1142 w	D13
1156 s	1158 w	1163 w	D13
1248 s	1249 m		D11
	1412 sh	1414 vw	D8
1432 m, vbr	1431 s/1437 sh	1439 vw, br/1441 vw	D7
	1452 w	1451 vw	D7
	1552 w/1563 vw	1555 w/1565 m	D5
	1572 vw	1573 w	D6
	3048 vw	3048 m	
	3065 vw, 3072 vw	3066 w	

^a^ The mode number in the Wright *et al*. assignment scheme ([30], see also electronic supplementary material, figure S3).

^b^ INS at 10 K.

^c^ Infrared at room temperature.

^d^ FT-Raman at room temperature. Values in brackets are dispersive 10 K data. Above 200 cm^−1^ the difference between room temperature and 10 K data is, at most, a few cm^−1^.

br, broad; m, medium; s, strong; sh, shoulder; v, very; w, weak.

**Figure 6.**
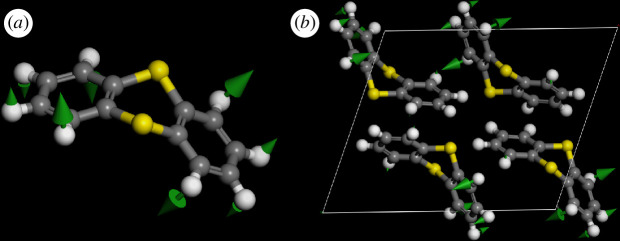
Comparison of thianthrene out-of-plane C–H bending mode D24 at ~860 cm^−1^ in (*a*) the isolated molecule and (*b*) the solid state.

Inspection of the mode animations shows that the *ortho*-phenylene modes largely conform to the archetypes shown in electronic supplementary material, figure S3, and the spectra are similar to those of centrohexaindane, which contains six *ortho*-phenylene groups coordinated to a central neopentane-like core [31]. However, there is one crucial difference between the idealized isolated molecule modes shown in electronic supplementary material, figure S3, and those present in the solid state; this is illustrated in figure 6.

**Figure 7.**
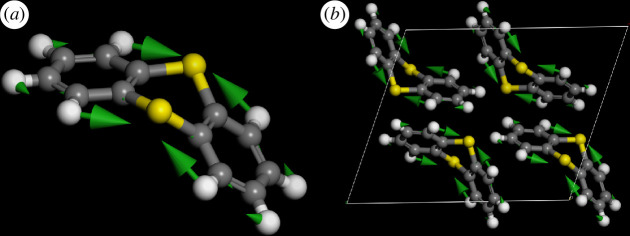
Comparison of thianthrene C–C stretch mode D7(1) at 1439 cm^−1^ in (*a*) the isolated molecule and (*b*) the solid state.

In thianthrene, the presence of two rings means that each mode in the isolated molecule (i.e. as shown in electronic supplementary material, figure S3) has two components with the rings moving either in-phase or out-of-phase with each other. In figure 6*a*, the two rings are moving out of phase with equal amplitudes. In figure 6*b*, the two rings are also moving out of phase but the amplitudes are very different. This is because in the solid state, the symmetry of the mode is for the entire unit cell; it is not restricted to a single molecule, as is the case in the isolated molecule situation. Curiously, this does not happen for every mode: figure 7 shows an example where the isolated molecule and solid-state modes are essentially identical. On detailed inspection, it is apparent that all of the C–H stretch modes are asymmetric (one ring has larger displacements than the other), as are most, but not all, of the modes in the 1600–1000 cm^−1^ range. Below 1000 cm^−1^, most of the internal modes are symmetric (both rings have equal atomic displacements). We note that this is roughly the dividing line between in-plane and out-of-plane modes, although in both regions there are exceptions. The distinction is not absolute; there are several modes, for example, 1423, 1121, 732 cm^−1^, where the atomic displacements are similar, but not identical, in amplitude in both rings.

A possible cause of the different behaviour of the modes is that the lateral and parallel interactions are different. The solid-state structure of thianthrene is described as [32]: ‘consists of puckered and interleaved layers of thianthrene molecules’ and is shown in electronic supplementary material, figure S4. It can be seen that the molecules are arranged end-to-end within a layer, and adjacent layers are nearly perpendicular to each other. Thus, there are no π–π stacking-type parallel interactions, so distinguishing parallel and lateral interactions is not possible. We note that the X-ray study stated [32]: ‘The closest approach between molecules occurs between two carbon atoms separated by 3.61 Å. The shortest distance between sulfur atoms in adjacent molecules is 3.77 Å; the shortest intermolecular S–C contact is 3.83 Å’. Thus, all the intermolecular interactions are weak. This is supported by the similarity of the dihedral angle in the gas phase, 131° [5], and the solid state, 128° [7]. In solution, where the interactions are stronger, this increases to 142° [6]. It appears that the intermolecular interactions are not the cause of the differing behaviours of the isolated and solid-state systems.

In contrast to the extensively studied *ortho*-phenylene modes, modes involving C–S have been little studied, particularly for aromatic systems [29]. The presence of two sulfur atoms results in six modes: four stretches and two bends. The individual C–S stretches and C–S–C bends combine as shown in table 2.

**Table 2.** Schematic of the six thianthrene modes that involve sulfur. For the C–S stretch modes: **+** = C–S bondlength increase, **−** = C–S bondlength decrease. For the bending modes: **+** = C–S–C bond angle increase, **−** = C–S–C bond angle decrease.

C–S stretch modes				C–S–C bend modes	
1	2	3	4	5	6
**+ −**	**+ +**	**+ +**	**+ −**	**+**	**+**
**+ −**	**− −**	**+ +**	**− +**	**−**	**+**

These are not pure modes, that is, involving only the C–S bonds, but will contribute to the *ortho*-phenylene modes. This is apparent from electronic supplementary material, figure S3, where some of the modes involve significant sulfur motion.

**Figure 8.**
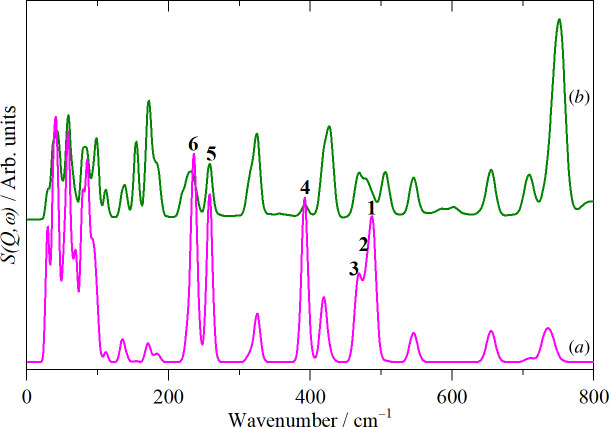
Calculated INS spectra of thianthrene: (*a*) modes that involve significant motion of sulfur and (*b*) all-atom contributions included. **1–4** denote the four C–S stretching modes and **5, 6** the two C–S–C bending modes shown in table 2.

The modes can be located in a pseudo-INS spectrum that emphasizes modes that involve the motion of sulfur. As the INS spectrum is purely dynamic, by setting the cross-section of all the atoms except sulfur to zero, only modes that have sulfur motion will have any intensity. Figure 8 shows a comparison between the ‘all-atom’ and the S-only INS spectra. Note that the S-only spectrum has been ordinate expanded by ×1000. This is a consequence of the small cross-section of sulfur and its significant mass. The former means that the bands are intrinsically weak, and the latter that the amplitude of motion is small. In combination, these two factors mean that the contribution of sulfur to the observed intensity is small.

There are no modes above 800 cm^−1^ that have any significant sulfur contribution. The literature [29] states that C–S stretch modes occur in the range 600–800 cm^−1^, there is no mention of the bending modes. From figure 8, ignoring the translational and librational modes below 100 cm^−1^, there are 12 modes with significant intensity in the 100–800 cm^−1^ region. It is striking that the modes with the largest intensity are not those in the 600–800 cm^−1^ range.

**Figure 9.**
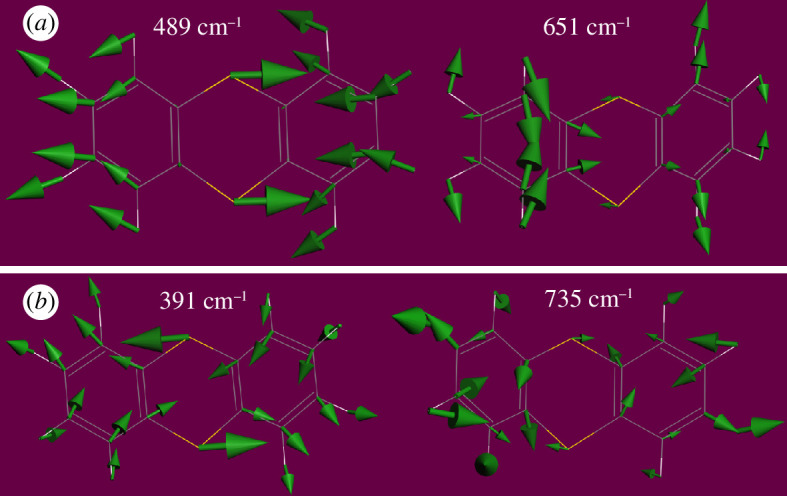
Atom displacements of thianthrene that involve significant motion of sulfur: (*a*) modes with C–S stretches of 1 of table 2) and (*b*) modes with C–S stretches of 4 of table 2.

Inspection of the mode animations is informative. Figure 9*a* shows a comparison of the modes at 489 and 651 cm^−1^ and figure 9*b* shows those at 391 and 735 cm^−1^. These are candidates for modes 1 and 4, that are shown schematically in table 2. In figure 9*a,b*, the high energy mode falls into the accepted range for C–S stretch modes. However, in both cases, it is apparent that the sulfur motion is a consequence of the *ortho*-phenylene mode (D17 and D16), respectively (electronic supplementary material, figure S3). In contrast, for 489 and 391 cm^−1^, it is the C–S stretches that are driving the mode. Modes **2** and **3** are located at 476 and 462 cm^−1^, respectively; these are illustrated in electronic supplementary material, figure S5.

For the two bending modes, 5 and 6, the obvious candidates are the modes at 257 and 235 cm^−1^ as these are the modes with the largest sulfur displacements. Inspection of the mode animations confirms this suggestion. The higher energy mode is the out-of-phase bending mode, and the lower energy is the in-phase (butterfly) mode. These are illustrated in electronic supplementary material, figure S6.

## 4. Conclusions

In this work, we have carried out a comprehensive characterization of the vibrational spectroscopy of thianthrene. The combination of infrared, Raman and INS spectroscopies is highly complementary and allows all of the modes to be observed. Periodic DFT calculations have provided unambiguous assignments of the spectra.

Thianthrene can be considered to be two *ortho*-phenylene units linked by sulfur atoms. The vibrations of the *ortho*-phenylene units largely conform to expectations; however, the presence of four molecules in the primitive cell means that the relative amplitudes of the atomic motions in each ring are not required to be equal. We find that approximately half the modes have equal (or nearly so) amplitudes in both rings. Generally, modes below 1000 cm^−1^ are symmetric (equal amplitudes in both rings), and above 1000 cm^-1^ are asymmetric (unequal amplitudes in both rings), but there are exceptions in both regions. The reason(s) for this are not apparent to us.

The literature states that C–S stretch modes occur in the 600–800 cm^−1^ range. We find that while there are modes that involve sulfur motion in this region, this is a consequence of motion in the *ortho*-phenylene rings. The modes that are driven by the C–S stretches are found in the ~400–500 cm^−1^ range. The C–S–C bending modes occur in the 200–300 cm^−1^ range; these have not been previously characterized. One note of caution is that the literature [29] values of 600–800 cm^−1^ for the C–S stretches are largely based on aliphatic materials, whereas the present work is for an aromatic system, and this may account for the differences.

## Data Availability

The datasets supporting this article are available from the Science and Technology Facilities Council data repository eData [33]. Supplementary material is available online [34].
